# Multiparametric Monitoring of Hypnosis and Nociception-Antinociception Balance during General Anesthesia—A New Era in Patient Safety Standards and Healthcare Management

**DOI:** 10.3390/medicina57020132

**Published:** 2021-02-02

**Authors:** Alexandru Florin Rogobete, Ovidiu Horea Bedreag, Marius Papurica, Sonia Elena Popovici, Lavinia Melania Bratu, Andreea Rata, Claudiu Rafael Barsac, Andra Maghiar, Dragos Nicolae Garofil, Mihai Negrea, Laura Bostangiu Petcu, Daiana Toma, Corina Maria Dumbuleu, Samir Rimawi, Dorel Sandesc

**Affiliations:** 1 Faculty of Medicine, “Victor Babes” University of Medicine and Pharmacy, 300041 Timisoara, Romania; alexandru.rogobete@umft.ro (A.F.R.); bedreag.ovidiu@umft.ro (O.H.B.); marius.papurica@gmail.com (M.P.); claudiu_barsac@yahoo.com (C.R.B.); andramaghiar@yahoo.com (A.M.); dsandesc@yahoo.com (D.S.); 2 Anaesthesia and Intensive Care Research Center, “Victor Babes” University of Medicine and Pharmacy, 300041 Timisoara, Romania; daiana.toma@yahoo.com (D.T.); corina.maria.d@gmail.com (C.M.D.); 3 Clinic of Anaesthesia and Intensive Care, Emergency County Hospital “Pius Brinzeu”, 300723 Timisoara, Romania; rimawi.samir@gmail.com; 4 Department of Vascular Surgery, “Victor Babes” University of Medicine and Pharmacy, 300041 Timisoara, Romania; rataandreealuciana@gmail.com; 5 Clinic of Vascular Surgery, Emergency County Hospital “Pius Brinzeu”, 300723 Timisoara, Romania; 6 Faculty of Medicine, “Carol Davila” University of Medicine and Pharmacy, 020021 Bucharest, Romania; dragosgarofil@gmail.com; 7 Faculty of Political, Administrative and Communication Sciences, Babes-Bolyai University, 400376 Cluj Napoca, Romania; negrea.mihai@gmail.com; 8 Faculty of Management, The Bucharest University of Economic Studies, 020021 Bucharest, Romania; laurabostangiu@yahoo.com

**Keywords:** hypnosis, multimodal monitoring, entropy, qNOX, qCON, bispectral index, surgical plethismographic index, general anesthesia, patient safety

## Abstract

The development of general anesthesia techniques and anesthetic substances has opened new horizons for the expansion and improvement of surgical techniques. Nevertheless, more complex surgical procedures have brought a higher complexity and longer duration for general anesthesia, which has led to a series of adverse events such as hemodynamic instability, under- or overdosage of anesthetic drugs, and an increased number of post-anesthetic events. In order to adapt the anesthesia according to the particularities of each patient, the multimodal monitoring of these patients is highly recommended. Classically, general anesthesia monitoring consists of the analysis of vital functions and gas exchange. Multimodal monitoring refers to the concomitant monitoring of the degree of hypnosis and the nociceptive-antinociceptive balance. By titrating anesthetic drugs according to these parameters, clinical benefits can be obtained, such as hemodynamic stabilization, the reduction of awakening times, and the reduction of postoperative complications. Another important aspect is the impact on the status of inflammation and the redox balance. By minimizing inflammatory and oxidative impact, a faster recovery can be achieved that increases patient safety. The purpose of this literature review is to present the most modern multimodal monitoring techniques to discuss the particularities of each technique.

## 1. Introduction

The rapid developments in the field of anesthesia, including new drugs, new anesthetic techniques, and new monitoring devices, have led to an increased trust in the anesthetic act from the general population and increased addressability toward surgical services, also promoting the development of more complex surgical techniques. In order to keep up with the demand multiparametric monitoring techniques in general anesthesia, rapid adaption is needed. This would lead to shorter waiting times, less post-operatory adverse events, and an increase in patient safety [1,2,3,4,5,6,7,8,9].

The state of consciousness is represented by a series of variables that can be experienced and felt, such as perceptions, sensations, emotions, and memories, making the quantitative analysis of these states impossible. One of the first state-of-consciousness theories launched in 1949 by Hebb, who postulated that the physical transposition of a mental representation is given by the neuro-cellular assembly and by the neuronal interconnections [5]. The N-metil-D-aspartate (NMDA) synapses were discovered based on this first theory, and after numerous studies, researchers found that synapses are predominantly found in the cortex [6,7,8]. Diverse interactions, ionic exchanges, the production of nitric oxide, and the electrical stimulation generated by the opening and closing of ion channels leads to the formation of inter-neuronal connections and to a complex neuronal activity. The loss of consciousness can have a number of causes, such as anesthesia, cerebral lesions, or sleep. In the case of anesthesia, the responses of the central nervous system are totally suppressed. This state is reversible, and it is an attribute of the development of modern medicine that has enabled the development of modern surgery and invasive therapeutic and diagnostic techniques [10,11,12,13,14,15,16,17,18,19,20,21,22,23,24,25].

Multimodal monitoring techniques in general anesthesia refer to the utilization of all parameters that characterize this process. Therefore, we talk about monitoring of the degree of hypnosis, of the nociception-antinociception balance, and of the functionality of the autonomic nervous system [23]. In the classical state of things, general anesthesia monitoring includes the evaluation of vital functions such as heart rate, blood pressure, temperature, and other subjective clinical findings. In this situation, there is always a risk of under- or overdosage of anesthetics, leading to either awareness or an excessive degree of hypnosis, with serious impact on the outcome and prognosis of these patients. Clinical signs such as hypertension, tachycardia, and tearing have long been used for guiding general anesthesia, but it has already been proven that they are subjective and cannot guide general anesthesia in an individualized manner, in accordance with the real needs of each patient [2,24,25].

Electroencephalography (EEG) is the recording of postsynaptic potentials in the pyramidal cells of the cerebral cortex. EEG is classified then based on the frequency. It can be recorded on the scalp and forehead using surface electrodes and reflects the metabolic activity of the brain. The metabolic activity of brain cells needs energy. Problems or changes in energy production (increased demand or decrease production) by brain cells can profoundly affect EEG activity [10,11,12]. Monitoring of the level of consciousness during general anesthesia based on electroencephalography has become a routine practice in the operating room. Both for the patient and anesthetist, the main concern during general anesthesia is the state of unconsciousness, mainly avoiding the risk of awareness. EEG models are well known to change in pattern with the deepening of anesthesia. Therefore, evaluating the degree of hypnosis requires measurements of the electrical activity of the brain [13,14,15]. The brain is the target effect site of anesthetics. Therefore, the brain needs to be monitored together with spinal reflexes and cardiovascular changes such as mean arterial pressure and heart rate. Measuring the depth of anesthesia is based on continuous EEG monitoring. Certain algorithms have been developed able to translate changes in the EEG signals into simple numerical indices that correspond to a certain level of anesthesia, from awake state to deep sleep [3,16,17]. Monitoring the state of consciousness is a complex endeavor and, although this clinical area has evolved rapidly, the benefits of EEG monitoring-based anesthesia are still controversial. The problem lies in the fact that our understanding of the human consciousness state is still incomplete, and we still lack information about the exact effects of general anesthesia on the brain. The depth of anesthesia is neither stable nor constant. Instead, it is more of a dynamic action that depends on the balance between the dosage of anesthetic medication and the pain caused by the surgical intervention [18,19,20].

Using EEG signal in order to monitor the depth of general anesthesia should reduce the incidence of intra-anesthetic awareness, lead to a reduction in anesthetic medication consumption, reduce the incidence of adverse effects related to anesthesia, and lead to shorter recovery times [21,22].

## 2. Multimodal Monitoring Techniques for the Degree of Hypnosis

In the current clinical practice, achieving an individualized prediction of the response to sedation and hypnosis is not accurate without multiparametric monitoring because of complex factors and variables that interfere with the anesthetic act. Among these, the most common are the concomitant administration of anesthetic agents, as well as the different pharmacokinetic response and the individual pharmacodynamic variability. Therefore, real-time monitoring of the effects induced by general anesthesia can bring an important contribution to the optimization of anesthetic dosage and hemodynamic control by the individualized titration of these medications. In order to limit perioperative adverse effects induced by the anesthetic drugs, it is recommended to titrate the doses based on the individual clinical response [26,27,28]. Some of the most common techniques for the evaluation and quantification of the degree of hypnosis are represented by the bispectral index (BIS, Medtronic-Covidien, Dublin, Ireland), Entropy (GE Healthcare, Helsinki, Finland), composite auditory evoked potential index (cAAI, AEP Monitor/2, Danmeter A/S, Odense, Denmark), and Narcotrend index (NCT, MonitorTechnik, Germany).

Bispectral analysis is a statistical technique that reveals nonlinear phenomena such as the electroencephalogram (EEG). The conventional analysis of EEG signals using standard procedures can bring important information regarding the frequency, power, and phase of EEG signals. The bispectral analysis of these signals represents a separate technique that analyses sinusoidal segments of the EEG spectrum, showing quantifiable variables in the form of an index (BIS) with clinical applicability. From a practical viewpoint, BIS is represented by a numeric interval between 0 and 100, where 0 represents the complete electrical abolition translated through cortical suppression and 100 is characterized by the conscious (awake) state on the EEG [29].

Another technique used for monitoring and individualizing the degree of hypnosis in patients undergoing general anesthesia is Narcotrend (Monitor Technik, Bad Bramstedt, Germany). Narcotrend is based on analyzing the EEG signal, and it classifies the degree of hypnosis in different levels, such as A (awake) and F (electrical silence), quantified by the Narcotrend Index, which ranges from 100 (awake) to 0 (electrical silence) [30]. In a study that compared the performance of the BIS and Narcotrend Index, Kreuer et al. reported similar effects of the two techniques. This research group obtained a prediction probability, P(K), for Narcotrend of 0.88 ± 0.03, while the P(K) for BIS was 0.85 ± 0.04. Furthermore, the mean drug effect, k(e0), was 0.2 ± 0.05 min^−1^ for Narcotrend and 0.16 ± 0.07 min^−1^ for BIS [31]. A similar study was carried out by Kreuer et al., who also reported similarities between the two techniques. Their study included 50 patients undergoing orthopedic surgery and reported statistically significant correlations between the D and E segments of Narcotrend and the 64–40 range of BIS [32]. Another study on the impact of hypnosis monitoring by Narcotrend Index in the pediatric patient population reported strong correlations between the Narcotrend Index and the minimum alveolar concentration (MAC) in patients over 4 months of age [33].

The Auditory evoked potentials (AEPs) represent another technique used for monitoring the degree of hypnosis in patients under general anesthesia [34]. Mantzaridis et al. studied the AEPs Index in patients undergoing orthopedic surgery. The mean value for the index at the beginning of surgery was 72.5 ± 11.2, followed by a decrease to 39.6 ± 6.9 that correlated with loss of consciousness. After recovery from anesthesia, the mean value for the AEPs Index was 66.9 ± 12.5, leading to the conclusion that this index is suitable for being used in the current medical practice [35].

On the other hand, the concept of Entropy derives from thermodynamics and is successfully used in the current clinical practice, with applications in EEG signal analysis. Regarding the mechanism of analysis, the EEG signal is first analyzed based on the “Fast Fourier” [28,29,30,31] concept for the identification of the sinusoidal compounds. After identifying the spectra, the Shannon function is applied in order to identify the specific values for each identified frequency. The sum of these values results in the numerical values called Spectral Entropy. The first algorithm ever to be used in the clinical practice has been defined and applied in the M-Entropy modules S/5 (GE Healthcare, Helsinki, Finland) [10,29,30,31,32,33,34,35,36,37,38,39,40,41,42,43,44,45]. The EEG data are collected through an adhesive sensor made of three electrodes applied on the fronto-temporal region. Applying this concept for general anesthesia led to the idea that when the brain is in the “awake status,” the EEG signals are complex and present, with a high degree of irregularity. When the patient is asleep/under general anesthesia, the neuronal activity progressively decreases, and the EEG complexes become more regular. Applying the principle in the case of Entropy in patients under general anesthesia, a significant difference has been observed regarding the wave spectrum generated, with this wave spectrum being directly proportional with the neuronal activity. Because the EEG signals are measured from electrodes placed on the frontal region, a high number of signals are represented by the activity of the muscles from the forehead region and are translated though an electromyography signal (EMG). Therefore, the EEG signals are defined by frequencies up to 32 Hz, while the EMG activity includes signals above 32 Hz. The M-Entropy module (GE Healthcare, Helsinki, Finland) distinguishes these two frequencies and generates two different parameters, both having important clinical significance—State Entropy (SE) and Response Entropy (RE). SE (0.8–32 Hz) reflects the cortical status of the patient, while RE (0.8–47 Hz) includes both the EEG and the EMG activity [12,34,35,36]. The values of SE are between 0 (suppressed EEG) and 91 (“awake status”), while RE is characterized by values between 0–100. In clinical practice, it is recommended to maintain RE/SE between 40 and 60 in order to achieve an adequate degree of hypnosis. Spectral Entropy is based on the analysis of frontal EEG and EMG variations and is a safe and reliable method for monitoring the depth of anesthesia. The Entropy module transforms the irregular content of the EEG signal in an index that reflects the depth of anesthesia. Normally, the signal is acquired from the skin on the forehead and temporal. Hence, it encompasses both an EEG and an EMG component [37]. The index is then calculated based on the following: High levels of entropy during anesthesia demonstrate awareness, while very low entropy levels are correlated with a profound state of unconsciousness. Using this parameter will lead to a more rapid awakening of the patient at the end of surgery and a lower dosage of anesthetic drugs. At the same time, it will prevent intra-anesthetic awareness episodes [32,38,39,40].

Changes in neuronal activity can be analyzed indirectly through computed tomography with integrated positron emission (PET-CT). This analysis is based on the changes in certain variables, such as neuronal activity, cerebral blood flow, and cellular metabolism [41]. Thus, specific changes in the glucose metabolism rate and cerebral blood flow can be quantified using [^18^F]–fluorodeoxiglucose and [^15^O] H_2_O. General anesthetic agents such as sevoflurane and propofol reduce the cerebral blood flow, with this effect being more important in the case of propofol. Maksimow et al. carried out a study regarding the changes in neuronal activity under general anesthesia and mapped the cerebral areas that better correlated with the EEG signals. The analysis of the regional cerebral blood flow was studied at different degrees of hypnosis measured by the Minimum Alveolar Concentration (MAC). In particular, the authors used MAC:1, MAC:1.5, and MAC:2 for sevoflurane, and different half maximal effective concentrations for propofol (EC50) at 30 min intervals. For patients in the sevoflurane group, the authors analyzed the End-Tidal Sevoflurane (Et-Sevo): 0% Et-Sevo (patient awake), 2% Et-Sevo (1 MAC), 3% Et-Sevo (1.5 MAC), and 4% Et-Sevo (2 MAC), while for the propofol group the analyzed group, the authors measured 0 microg/mL (patient awake), 6 microg/mL (1 EC50), 9 microg/mL (1.5 EC50), and 12 microg/mL (2 EC50). In both groups, the Entropy was inversely proportional with the sevoflurane and propofol concentrations, with reductions from 73.5 ± 6.5 to 12.2 ± 9.4 and from 70.4 ± 7.1 to 0.6 ± 1, respectively, in the frontal region. In the temporo-occipital region, the Entropy analysis was similar, following the same dose-dependent trend. Regarding the correlation between EEG/SE analysis and computed tomography, the researchers found statistically significant correlations for both drugs at similar concentrations (1.5 MAC, *r* = 0.81 și 1.5 EC50, *r* = 0.83)**.** Following this study, Maksimow et al. validated the fact that spectral Entropy can be used for both sevoflurane and propofol, showing the same regional neuronal activity confirmed through noninvasive PET-CT analysis. The usage of monitoring techniques for the degree of hypnosis in the case of pediatric patients is limited and is not validated. Numerous studies have analyzed the statistical correlations between BIS and Entropy for different age groups but have not identified strong statistical correlations between BIS/Entropy values and anesthetic drugs concentrations in infants vs. pediatric patients (aged over 1 year old) [42]. Davidson et al. carried out a study regarding the performance of BIS and Entropy for different age groups in pediatric patients. They analyzed four age groups: 0–1 years old (*n* = 8), 1–2 years old (*n* = 10), 2–4 years old (*n* = 18), and 4–12 years old (*n* = 14). Regarding the comparison between Entropy and BIS, above the initial status (awake), they identified statistically significant differences in the 0–1 years old group, as follows: RE/BIS: 45 vs. 84, *p* = 0.003, SE/BIS: 36 vs. 78 (*p* = 0.02). Following this study, no statistically significant differences have been proven for BIS or for Entropy, especially in the 0–1 age group. Interestingly, there were no performance differences between BIS and Entropy but applying these techniques in the case of infants should be done with caution. In Table 1, a series of implications for different monitoring techniques for the degree of hypnosis on the clinical prognostic of patients undergoing general anesthesia are summarized [43].

One other widely discussed risk is the incidence of intraoperative awareness that can lead to long-term posttraumatic stress disorder. Sebel et al. carried out a study on the incidence of intra-anesthetic awareness analyzing 19,575 patients. They identified 25 patients that presented with awareness, resulting in an incidence of 0.13%. This research group did not find any statistically significant differences regarding the incidence based on sex or age, but increased incidence was associated with higher The American Society of Anesthesiologists (ASA) physical status classification system scores (odds ratio, 2.41; 95% CI, 1.04–5.60 ASA III–V vs. ASA I–II) [55]. Sebel et al. estimated a rough number of 26,000 cases of intra-anesthetic awareness annually in the United States, and this number is reported to be approximately 20 million among general anesthesia procedures [55]. In a similar study, Bruhn et al. reported an incidence of 0.11% out of 10,811 patients [34]. Ekman et al. reported a 0.18% incidence of awareness in a retrospective study that included 7826 patients [56]. For all listed studies, the incidence of awareness was lower in the groups of patients where techniques for monitoring the degree of hypnosis were used [34,55,56].

## 3. Monitoring Techniques for the Nociception-Antinociception Balance

**Figure 1:**
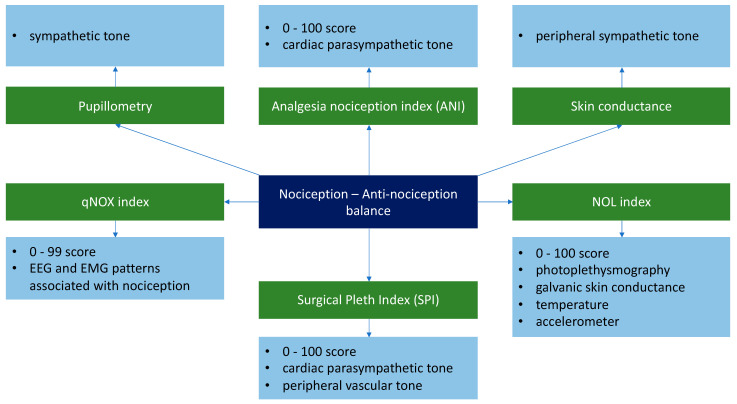
Technologies/parameters used for monitoring the nociception-antinociception balance [59,60,61,62,63,64,65,66]. ANI—analgesia nociception index; qNOX index—index of nociception; SPI—Surgical Plethysmographic Index; NOL index—Nociception Level Index; EEG—Electroencephalography; EMG—Electromyography signal.

Another important aspect in the clinical practice is represented by the continuous monitoring of the nociception-antinociception balance. The aim of these parameters is to aid the clinician in deciding the adequate analgesia dosage for each patient. Whereas monitoring the degree of hypnosis is achieved through the direct evaluation of the EEG signals, the nociception-antinociception balance can be monitored indirectly [9,12] by evaluating certain variables such as the vasomotor reflex, pupillary size, the H reflex, and the hemodynamic response [57,58] (Figure 1).

One of the most widely studied technologies is the analysis of hemodynamic changes, including the evaluation of the normalized heartbeat intervals (HBIs) and of the amplitude of the plethysmographic waves, both correlating with sympathetic and parasympathetic tones. A higher sympathetic tone correlates with the intensity of the surgical stimuli and results in a suppressed plethysmographic amplitude (PPGA). For the correct calculation of the Surgical Plethysmographic Index (SPI), after normalizing these parameters by transforming the histogram, the SPI formula is used where SPI = 100 − (0.67 × PPGA_norm_ + 0.33 × HBI_norm_). The SPI value can be influenced by certain factors, such as cardiac pacemakers, cardiac arrhythmias, antiarrhythmic medication, beta−1 adrenergic antagonists, and alpha2-adrenergic agonists. Bonhomme et al. evaluated the Surgical Pleth Index (SPI, GE Healthcare, Helsinki, Finland) trend and made correlations with variability in mean arterial pressure and heartrate. Following this study, they showed that there is a strong correlation between all these variables. Therefore SPI values depend on the doses of opioid medication administered during the anesthesia [58]. Bergmann et al. carried out a randomized study that included 170 patients receiving general anesthesia with propofol and remifentanil. The patients were divided in two study groups. One study group received opioids based on SPI values, while the other group received the doses of opioids based on standard monitoring parameters, both clinical and hemodynamic monitoring. Statistically significant differences were shown in both propofol (*p* < 0.05, 6.0 ± 2.1 vs. 7.5 ± 2.2 mg/kg/h) and remifentanil (*p* < 0.05, 0.06 ± 0.04 vs. 0.08 ± 0.05 μg/kg/min) consumption. The impact on post-anesthesia recovery time was evaluated by the time needed to open the eyes and time to extubation. The results presented statistical significance for both the evaluated features, extubation time (*p* < 0.05, 1.2 ± 4.4 min vs. 4.4 ± 4.5 min), and eye-opening time (*p* < 0.05, −0.08 ± 4.4 min vs. 3.5 ± 4.3 min). The conclusion was that dose reduction and shorter recovery times can be achieved by adapting general anesthesia based on the SPI [10]. Huiku et al. confirmed in a similar study that SPI monitoring has a beneficial impact on anesthetic drugs used doses, increasing patient safety and the quality of the medical services [67].

Another parameter used for the evaluation of the nociception-antinociception balance is the Analgesia Nociception Index (ANI) [68]. The technology is based on the assessment in heart rate variability. In the clinical setting, ANI values lie between 0 and 100. In this case, 0 represents a very low degree of parasympathetic modulation and 100 represent a very high degree of parasympathetic activity. From a clinical point of view, ANI = 0 represents high stress levels, while ANI = 100 represents low stress levels.

Dostalova et al. carried out a study in which they compared the impact the two monitoring techniques have on general anesthesia. They had three study groups: The group where doses of opioids were titrated based on ANI values, the SPI group, and the control group. They showed statistically significant differences regarding the decrease in opioid consumption and shorter recovery times after anesthesia [68]. Table 2 summarizes a series of studies regarding the impact of monitoring techniques on the nociception-antinociception balance and on the clinical outcome of patients.

Numerous studies have shown that opioid overdose during anesthesia is responsible for a series of adverse effects, such as increased recovery times and opioid induced hyperalgesia, and that opioid overdose also leads to hypotension, having a major impact on perioperative hemodynamic stability [66,67,68,69,70,71]. Won et al. reported that using SPI monitoring during general anesthesia reduced opioid consumption, improved hemodynamic stability, and reduced postoperative recovery times [71]. A similar study was carried out by Jain et al., which showed a statistically significant decrease in the number of hemodynamic adverse events when SPI was used for the titration of opioid medication (*p* < 0.05) [70].

Another system used for monitoring the nociception-antinociception balance is the index of nociception (qNOX) (qCON 2000 Monitor, Quantium Medical, Fresenius Kabi, Mataro, Spain). This parameter is based on the evaluation of EEG and EMG patterns, with values between 0 and 99. Jensen et al. carried out a study on 60 patients undergoing general anesthesia with propofol and remifentanil and showed a series of statistically significant correlations, concluding that qNOX can detect fine changes in the nociception-antinociception balance [76]. The Nociception Level Index (NOL Index, Medasense, Ramat Gau, Israel) is another widely used technology for titrating analgesic drugs during general anesthesia. It analyses the photoplethysmographic wave, temperature, skin galvanic conductance response, and accelerometry [63].

## 4. The Impact of Multimodal Monitoring on the Hemodynamic Status

During general anesthesia, maintaining adequate tissue perfusion represents one of the most important goals in the perioperative management of the patient. Hypotension frequently occurs, especially after the induction of anesthesia, that is, between the moment of induction and the start of surgery. Reich et al. reported a decrease in mean arterial pressure (MAP) of over 40% (MAP < 70mmHg or MAP < 60 mmHg) in the first 10 min after induction (*p* < 0.001) [77]. Moreover, this study (*n* = 2406 patients) reported an increase in the time spent in the recovery room (13.3%, *p* < 0.05) and in postoperative mortality rates (8.6%, *p* < 0.02) in patients that presented perioperative hypotension. Another interesting phenomenon presented by the group was that post-induction hypotension was more frequent in the 5–10 min interval in comparison to the 0–5 min interval after induction of general anesthesia [77]. A similar study carried out by Hug et al. reported that over 15% of patients presented a decrease in systolic blood pressure (SBP) under 90 mmHg after induction with propofol in the first 10 min after administration [78]. Studies have shown that induction with sevoflurane maintains hemodynamic stability and decreases the risk of hypotension in comparison to induction with propofol, as this technique is not as well tolerated by the patients. Thwaites et al. studied the satisfaction of patients regarding the induction technique used: Sevoflurane (inhalational induction, 8%) vs. propofol (i.v. induction). Over 14% of the patients considered inhalational induction unpleasant in comparison to 10% in the case of propofol. Furthermore, over 24% of the patients would not choose sevoflurane induction the second time [79].

Cerebral ischemia is one of the main causes for cognitive impairment, with a very high global degree of mortality, while motor and cognitive dysfunctions seriously affect the quality of life of these patients. Cerebral reperfusion after an ischemic episode can induce organ damage such as neurovascular injury, neuronal death, cerebral edema, and neuro-hemorrhagic changes. The most common cellular mechanisms involved are represented by apoptosis, inflammation, and excessive production of free radicals [80].

The impact of hypotension during general anesthesia on the postoperative outcome and on the development of postoperative adverse events has been widely studied. Intraoperative hypotension (IHO) is a common effect of general anesthesia and has been associated with an increased incidence of 1-year mortality after surgery [81,82].

The most important predictors for perioperative morbidity and mortality are the associated comorbidities, the determinants of the surgical procedure, and the specific aspects of perioperative management and of general anesthesia. Apart from monitoring the hemodynamic parameters, quantification of the degree of hypnosis “depth of anesthesia” represents one of the most important parameters in modern general anesthesia. At the time, monitoring the degree of hypnosis is possible using techniques based on the analysis of electroencephalography signals (EEG) [83].

Monk et al. studied the 1-year prognosis of patients that underwent noncardiac surgery under general anesthesia. The research group carried out complex statistical analysis in order to determine if death at 1 year after can be associated with significant clinical features of the patient or with the management of general anesthesia. In order to control the degree of hypnosis, they used the Bispectral Index ^®^ (BIS^®^), with the same type of electrodes for all patients included in the study (A1050BIS Monitor, BIS sensors, Aspect Medical Systems, Newton, MA).

Global mortality at 1 year was 5.5% (*n* = 1604) and 10.3% for patients aged over 65 (*n* = 243). Regarding the variables that correlate with mortality, Monk et al. reported three statistically significant segments: 1. Patient comorbidities (relative risk 6.116, *p* < 0.05); 2. General anesthesia overdosage/deep anesthesia, BIS < 45 (relative risk 1.244/h, *p* < 0.05); 3. Systolic hypotension during surgery (relative risk 1.036/min, *p* < 0.05) [83]. They concluded that prolonged intraoperative hypotension can be associated with an increased incidence in mortality at 1 year [83]. Although numerous studies have focused on perioperative hypotension, at the time there, is no clear definition for IHO [84]. Most of the studies have addressed the statistical associations and correlations between different numerical intervals and correlations with the clinical changes. Sun et al. carried out a study on the impact of IHO on acute kidney injury (AKI). Furthermore, the research group investigated the implications IHO time have on the incidence of AKI. They correlated the AKI incidence with different IHO intervals as follows: MAP < 55 mmHg, MAP < 60 mmHg, and MAP < 65 mmHg [85]. This was a retrospective study that included 5127 patients between 2009 and 2012. The results showed an AKI incidence of 6.3% (324 patients) for MAP < 60 mmHg and an IHO time between 11–20 min, and MAP < 55 for an IHO time > 10 min. Sun et al. reported a strong statistical correlation between sustained episodes of IHO with a MAP < 50 mmHg and MAP < 60 mmHg and AKI incidence. For the evaluation of AKI, they considered a 50% increase in creatinine levels or 0.3 mg/dL in the first 2 days after surgery. A similar study was developed by Walsh et al. regarding the implications of IHO on the incidence of AKI and myocardial injury. They evaluated 33,330 patients that had undergone noncardiac surgery, making statistical correlations between the incidence of AKI and myocardial injury in patients that presented with IHO with a MAP < 55 mmHg and MAP < 75 mmHg. Following statistical analysis, they identified 2478 patients that had developed AKI (7.4%) and 770 (2.3%) with myocardial injuries. For both groups, MAP was under 55 mmHg. Interestingly, the risk for developing renal and myocardial lesions was increased, even for short IHO times [86]. In a similar context, a metanalysis carried out by Wesselink et al. reported ischemic organ damage when MAP < 80 mmHg for longer than 10 min. This research group showed an increase in risk with any decrease in blood pressure [84].

## 5. The Impact of General Anesthesia Multimodal Monitoring on Inflammation/Redox

Another important aspect that also has an impact on the clinical outcome of surgical patients is represented by the inflammatory status and the oxidoreduction response (REDOX) [87,88,89,90,91]. The excessive production of free nitrogen and oxygen radicals has a direct involvement in the augmentation of the pro-inflammatory status. Under physiological conditions, the balance between the production of free radicals and that of endogenous antioxidant substances maintains the oxidoreduction equilibrium and the body does not suffer. Under surgical stress, in the case of ischemia-reperfusion syndrome or hypotension, an excessive number of free radicals will be produced, as well as proinflammatory mediators. All these factors will also decrease the production capacity for antioxidant molecules [92].

**Figure 2:**
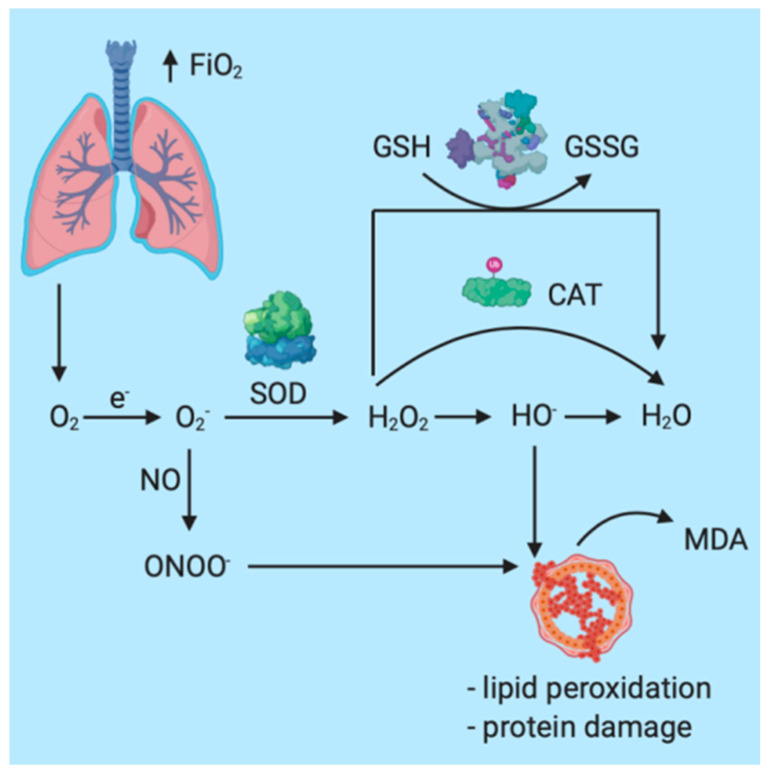
Schematic representation of the oxidative response in patients under general anesthesia.

Particularly in the case of patients under GA (general anesthesia) or in mechanically ventilated patients, oxygen plays an essential role in therapeutic management. In the case of general anesthesia, increased oxygen inspiratory fractions (FiO_2_) are administered before endotracheal intubation and after extubation in order to maintain an adequate oxygen plasma concentration. Under physiological conditions, P_a_O_2_ = 80–100 mmHg. When P_a_O_2_ exceeds 100 mmHg, the patient is characterized by hyperoxia, the most important systemic effect being the increased and accelerated production of reactive oxygen species (ROS) and the development of oxidative stress (OS) [93,94,95,96,97,98,99]. The most important mechanisms through which OS is augmented in the case of general anesthesia are represented by the increase in molecular oxygen offerings at the mitochondria, the interaction with reactive nitrogen species (RNS), and lipid peroxidation with destruction of cellular membranes [25,100,101,102,103] (Figure 2).

Nunes et al. studied the implications of general anesthesia (GA) on the redox profile of surgical patients that underwent intravenous GA, as well as the implications of multimodal monitoring based on Entropy on the oxidoreduction activity. The study included 20 patients divided into 2 study groups. In the first group, the Entropy values were maintained in the 45–59 interval, and in the second, Entropy was maintained in the 30–44 interval in order to evaluate the impact of anesthetic overdosage on the redox balance. The patients were evaluated at different moments in time: M1—right after the administration of anesthetic drugs, M2—after endotracheal intubation, M3—5 min after endotracheal intubation, M4—immediately after surgical pneumoperitoneum, M5—1 min after pneumoperitoneum, and M6—1 h after the end of surgery. The researchers determined the plasma concentrations for Glutathione and TBARS (tiobarbituric acid reactive species). Following the analysis, they identified significant increases in the Glutathione and TBARS concentrations at M5 in both groups. There were statistically significant differences between the two study groups, with higher values of both Glutathione and TBARS in the group where Entropy was maintained between 30 and 44 (*p* < 0.05). In regard to the anesthetic management, recovery times were significantly shorter for the group where Entropy levels were kept between 45 and 59 (7.70 ± 1.24 min vs. 10.20 ± 0.90 min, *p* < 0.05). The increase in redox imbalance markers for the patients that received a deeper hypnosis (Entropy 20–44) reveals an increase in anaerobic metabolism, possibly because of an accentuated suppression of the autonomic nervous system [92].

Ferrari et al. carried out a study regarding the genotoxicity of sevoflurane on the DNA structure in isolated lymphocytes in 20 patients undergoing orthopedic surgery under GA. They showed important changes in DNA structure and in redox activity that correlated statistically with the sevoflurane concentration [104]. Compared to the exposure to propofol, the group that was exposed to sevoflurane presented a marked increase in the expression of tumor necrosis factor alpha (TNF-alpha) and a decrease for interleukin 10 (IL-10) [104,105].

## 6. The Impact of Multiparametric Monitoring on Drug Consumption and Recovery

Gan et al. led an important study regarding the implications of monitoring the degree of hypnosis. They included 302 patients divided in 2 groups. In the study group, GA was guided based on BIS monitoring, while in the control group, anesthesia was guided with basic monitoring. BIS values were measured in both groups [106]. In the study group, the dosage of anesthetic agents was optimized in order to achieve a mean BIS value between 40 and 60 based on current guidelines and recommendations. Interestingly enough, the BIS values in the control group were under 40, indicating a tendency to overdose the anesthetic agents. The total propofol consumption was lower in the study group compared to the control. Another important variable was the time to extubation, which was 7.27 min shorter (95% CI 6.23–8.28 min) in the study group compared to 11.22 min in the control group (95% CI, 8.51–13.60 min). Song et al. designed a similar study that also showed a decrease in extubation times in patients that received general anesthesia modulated based on BIS, with a reduction from 6.5 ± 4.3 min to 3.6 ± 1.5 min (>40%) for Desflurane, and from 7.7 ± 3.5 min to 5.5 ± 2.2 min for sevoflurane [107].

Vakkuri et al. carried out a multicenter study on the impact the monitoring of degree of hypnosis through Entropy (GE Healthcare, Helsinki, Finland) has on anesthetic drug consumption and on postoperative recovery time. In the final analysis of the study, they included 308 patients, divided homogeneously in 2 groups: The control group and the study group, where GA was modulated based on Entropy. For propofol consumption, there were statistically significant differences between the two study groups, with the median for the control group being 0.11 (0.03, 0.21) mg/kg/min vs. 0.10 (0.04, 0.23) mg/kg/min for the group where Entropy was used.

The analysis of the implications multimodal monitoring has on the postoperative recovery showed a decrease in the time to spontaneous breathing in the study group (4.74 (0.00, 18.0) minutes) compared to the median in the control group (7.07 (1.00–28.5) minutes). Using Entropy also decreased the time to extubation from 9.16 (1.67, 32.3) minutes to 5.80 (3.00, 27.3) minutes, with *p* <0.05. The patients in the target group opened their eyes to verbal command faster than the control group (6.08 (0.15, 37.5) minutes vs. 10.8 (2.23, 43.2) minutes (*p* < 0.05)), and they were transferred to the Post-Anesthesia Care Unit (PACU) faster, at 10.3 (1.17, 48.7, *p* < 0.05) minutes vs. 13.0 (5.0, 49.8) minutes. Mean State Entropy (SE) during general anesthesia was 50 (34–78), while the mean Response Entropy (RE) was 52 (35–84). [46]. A similar study was developed by El Hor et al., reporting an increase in sevoflurane consumption in the case of patients that could not benefit from advanced monitoring of the degree of hypnosis vs. patients for which Entropy monitoring was applied (5.2 ± 1.4 mL/h vs. 3.8 ± 1.5 mL/h, *p* < 0.05) [108]. Regarding hemodynamic stability, the researchers found statistically significant differences between the groups: 10 hypertension episodes were reported in the control group vs. 7 hypertension episodes in the target group. For hypotension, the ratio was 3 in the control group vs. 0 in the target group (*p* < 0.05). Tachycardia episodes were reported as 5 (control group) vs. 8 (target group), while bradycardia episodes were reported as 1 (control group) vs. 0 (study group).

Wu et al. analyzed the impact of multiparametric monitoring based on Entropy (GE Datex-Ohmeda S/5) on the recovery time and anesthetic drugs consumption in patients undergoing orthopedic surgery. This research group included 68 patients in their analysis, divided into 2 groups: The target group with Entropy monitoring and the control group with classical anesthesia monitoring. Sevoflurane consumption was significantly lower in the target group 27.79 ± 7.4 mL/patient vs. 31.42 ± 6.9 mL/patient, *p* < 0.05. Statistically significant differences were also reported for hemodynamic stability, as the target group presented fewer hypertensive episodes compared to the control, 0.94 ± 1.15 vs. 1.48 ± 1.41, *p* < 0.05. Following this study, the research group concluded that using Entropy-based multimodal monitoring significantly reduces both sevoflurane consumption and the consumption of antihypertensive agents [45].

The impact of multiparametric monitoring on the anesthetic drugs consumption was proven in another study by Tewari et al. in patients undergoing gynecological and obstetrical surgery. They analyzed 120 patients that were divided into two study groups based on monitoring technique, with an Entropy group and a classical monitoring of general anesthesia group. They showed that Entropy monitoring led to a reduction of propofol doses (6.7% reduction, *p* = 0.01), but also that the Fentanyl doses were 10.9% larger in this group (*p* = 0.07). They did not find any statistically significant differences for recovery time and discharge time from PACU [109,110,111]. In their study on the impact of Entropy on sevoflurane consumption in major hepatic surgery, Refaat et al. showed a marked decrease in the doses [110].

## 7. Conclusions

General anesthesia techniques are much more advanced nowadays compared to latter decades, in accordance with the surgical needs and with the needs of the general population. Medical services tend to become more and more complex, managing to solve a wide range of pathologies in all surgical fields. In order to increase both patient safety and medical act quality, as well as to decrease waiting times and to be able to answer the needs of an increasing number of patients, endowment with modern multiparametric monitoring techniques for general anesthesia is necessary. In conclusion, we can state that using monitoring techniques for the degree of hypnosis, the nociception-antinociception balance, and the hemodynamic status markedly increases patient safety. Furthermore, by reducing postoperative recovery times and reducing anesthetic drugs doses, one can highlight the positive impact, both short- and long-term, that multiparametric monitoring has from an economic viewpoint.

## Figures and Tables

**Table 1:** The impact of monitoring the degree of hypnosis on anesthetic drugs consumption and on time recovery.

Author	Parameter/Monitoring Technique	Type of General Anesthesia	Observations	Reference
Kim et al.	State Entropy (SE)	78 children (age: 3–12) Sevoflurane	↓ sevoflurane consumption ↓ postoperative recovery time	[44]
Wu et al.	State Entropy (SE)	64 patients Sevoflurane	↓ sevoflurane consumption ↓ consumption of antihypertensive drugs ↑ hemodynamic stability	[45]
Vakkuri et al.	State Entropy (SE)	368 patients propofol-alfentanil-N_2_O	↓ propofol consumption ↓ postoperative recovery time	[46]
Talawar et al.	Entropy (SE/RE)	50 patients isofluran-N_2_O	↓ postoperative recovery time	[47]
Elgebaly et al.	Entropy (SE/RE)	propofol	↓ propofol consumption ↑ hemodynamic stability	[48]
Gan et al.	Bispectral index (BIS)	302 patients propofol-alfentanil-N_2_O	↓ propofol consumption ↓ postoperative recovery time	
Liu et al.	Bispectral index (BIS)	1383 patients Day surgery	↓ consumption of anesthetic drugs ↓ incidence of adverse effects (nausea, vomiting) ↓ postoperative recovery time	
Bhardwaj et al.	Bispectral index (BIS)	50 pediatric pts propofol	No effects have been observed regarding the consumption of anesthetic drugs No effects on the postoperative recovery time	[49]
Aime et al.	Bispectral index (BIS) and Entropy (RE/SE)	115 patients Sevoflurane;	BIS & Entropy: ↓ sevoflurane consumption	[50]
Liao et al.	Bispectral index (BIS) and A-line autoregressive index (AAI)	116 patients Sevoflurane;	BIS & AAI: ↓ sevoflurane consumption ↓ postoperative recovery time;	[51]
Weber et al.	Composite auditory evoked potential index (cAAI)	20 pediatric patients TIVA propofol and remifentanil;	↓ propofol consumption ↑ hemodynamic stability	[52]
Lai et al.	Narcotrend	40 patients propofol and fentanyl;	↓ propofol consumption ↓ postoperative recovery time No effects on PONV	[53]
Rundshagen et al.	Narcotrend	48 patients propofol and remifentanil	No effects on propofol/remifentanil consumption No effects on postoperative recovery time	[54]

RE: Response Entropy; SE: State Entropy; BIS: Bispectral Index; TIVA: Total Intravenous Anesthesia; PONV: Postoperative nausea and vomiting; AAI: A-line Autoregresive index; cAAI; composite auditory evoked potential index.

**Table 2:** The impact of nociception-antinociception monitoring techniques on anesthetic drugs consumption and on recovery time.

Author	Technique/Parameter	Type of Anesthesia Type of Intervention	Obervations	Reference
Funcke et al.	SPI & Pupillary Pain Index (PPI) & Nociception Level (NOL)	48 patients radical retropubic prostatectomy	SPI: ↓ hormonal response to stress PPI: ↓ sufentanil consumption, ↑ hormonal response to stress No effect on postoperative recovery time	[69]
Bergmann et al.	Surgical Pleth Index (SPI)	170 patients orthopedic surgery	↓ propofol consumption ↓ remifentanil consumption ↓ postoperative recovery time	[10]
Jain et al.	Surgical Pleth Index (SPI)	140 patients Laparoscopic cholecystectomy;	↓ PONV ↓ postoperative pain ↑ fentanyl consumption No impact on hemodynamic stability	[70]
Won et al.	Surgical Pleth Index (SPI)	45 patient; Elective thyroidectomy	↓ oxycodone consumption ↓ postoperative recovery time ↓ extubation time	[71]
Chen et al.	Surgical Stress Index (SSI)–former Surgical Pleth Index (SPI)	80 patients Elective surgical interventions	↓ remifentanil consumption ↓ postoperative adverse effects ↑ hemodynamic stability	[72]
Theerth et al.	Analgesia Nociception Index (ANI)	60 patients Oncological surgery	↓ fentanyl consumption No impact on postoperative pain	[73]
Soral et al.	Analgesia Nociception Index (ANI)	102 patients Procedural sedation	↓ opioid consumption No impact of propofol and ketamine consumption	[74]
Gall et al.	Analgesia Nociception Index (ANI)	60 patients Bariatric surgery	↓ sufentanyl consumption No impact on PONV and postoperative pain	[75]

## Data Availability

Not applicable.
